# Interventions to reduce cadmium exposure in low- and middle-income countries during pregnancy and childhood: A systematic review

**DOI:** 10.7189/jogh.12.04089

**Published:** 2022-11-12

**Authors:** Kam Sripada, Adrian Madsen Lager

**Affiliations:** 1 Centre for Digital Life Norway, Norwegian University of Science and Technology (NTNU), Trondheim, Norway; 2 Centre for Global Health Inequalities Research (CHAIN), Norwegian University of Science and Technology (NTNU), Trondheim, Norway; 3 Department of Chemical Engineering, Norwegian University of Science and Technology (NTNU), Trondheim, Norway

## Abstract

**Background:**

Exposure to the toxic metal cadmium is widespread globally and especially prevalent in low- and middle-income countries (LMICs). Early life (from pregnancy through childhood) is a vulnerable window for exposure. Therefore, interventions in low- and middle-income countries to prevent or reduce early life exposure to cadmium may be relevant for improving public health.

**Methods:**

We systematically reviewed five databases (Scopus, Web of Science, Global Health Medicus, Greenfile, and PubMed). A synthesis without meta-analysis (narrative synthesis) was used for data analysis due to the wide heterogeneity of included studies. Study quality and risk of bias were assessed using modified GRADE criteria.

**Results:**

4098 articles were returned by the search and a total of 26 studies from 21 LMICs were included in this review, ranging from policies to clinical treatment, rehabilitation and clean-up methods for agricultural soil, interventions for nutrition and cooking, and anti-pollution strategies at the household level. The interventions targeted children, pregnant and postpartum women, and/or women of childbearing age. While several studies provided some evidence of effectiveness, none appeared to offer a realistic solution for cadmium pollution at scale. Agricultural and food preparation studies were relatively frequent, particularly related to rice. Studies on air filtration during pregnancy indicated some effectiveness in reducing indoor cadmium exposures.

**Conclusions:**

Cadmium pollution is a persistent and widespread threat to children’s health with few identified solutions. Long-lasting damage to children’s health starting in the earliest years should motivate investment in higher-quality interventions, innovations, and further research.

**Registration:**

PROSPERO (CRD42021235435).

Children encounter the toxic metal cadmium frequently in early life. It is present in tobacco smoke, a variety of foods, air pollution, and consumer products like cheap jewellery, and some plastics. Cadmium is a human carcinogen (Group 1) [1] and is toxic to kidneys, skeletal and respiratory systems, and neurodevelopment [2-5]. Early life exposures to cadmium are of particular concern [3,6-9]. Globally, hundreds of millions of people are exposed to elevated cadmium beginning in early life [10] (**Figure 1**).

**Figure 1:**
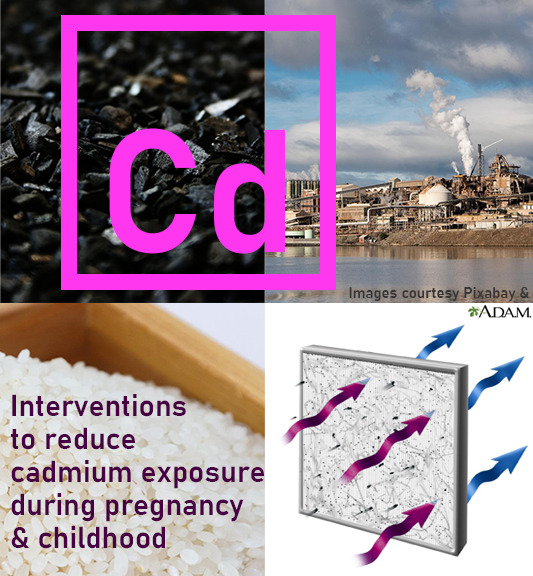
Graphical abstract for this study. Abstracts in Persian and Chinese can be found in file S1 in the **Online Supplementary Document**.

Low- and middle-income countries (LMICs) face a heavy burden of cadmium contamination due to industrialization [11-13]. Developing countries experience extensive uncontrolled cadmium release into the environment [14,15]. Food crops irrigated with cadmium-contaminated water are major sources of exposure [16]. Rice (*Oryza sativa*) is a staple crop for over half of the world’s population and provides more than 20% of the calories consumed globally [17]. It is also a known high-accumulator of cadmium from the environment, making it a major source of cadmium in LMICs [10,18-20]. Undernutrition and micronutrient deficiency has been found to increase cadmium uptake in pregnant mothers in LMICs [21-23]. Cadmium is commonly used in cheap jewellery and toys sold in LMICs such as Nigeria [24], Cambodia [25], and China [26], either made locally or imported. A study from China reported that cadmium exposure from food contributes substantially to stroke and coronary heart disease burden [27].

E-waste also contains cadmium (eg, nickel-cadmium batteries) and is transported in large quantities – often illegally – from high-income countries (HICs) to LMICs [28]. Major destinations for e-waste are Africa, Southeast Asia, Central America, and South America, where some components can be recycled [29]. Due to inadequate e-waste management infrastructure, hazardous e-waste materials frequently end up in landfills, open-air burning, or open dumping sites near residential areas [29]. Due to its toxicity, bioavailability, and soil concentration at e-waste dump sites, cadmium is considered a high environmental risk, especially for children [28,30]. At an informal e-waste recycling site in Lagos State, Nigeria, soil cadmium levels exceeded Dutch soil guideline values [30], along with elevated levels of antimony, chromium, copper, lead, manganese, nickel, and zinc.

Cadmium exposure is a global problem. Factories refining non-ferrous metals or recycling cadmium-containing scrap and e-waste, and waste incinerators (especially of cadmium-containing batteries and plastics) are sources of cadmium pollution [31-33]. Airborne particles containing cadmium can travel thousands of kilometres from their source [34]. Mining and drainage from mines (cadmium is a by-product of zinc mining) and waste disposal contaminates bodies of water. Foods grown in environments contaminated by cadmium from industrial emissions and runoff can also accumulate cadmium, such as grains, vegetables, meat, and seafood [33]. Tobacco leaves also naturally accumulate relatively high concentrations of cadmium [35]. Sewage sludge, manure, and some fertilizers may leach cadmium into agricultural soil [33]. Contaminated roadside soils are frequently used for growing food crops [36]. In many areas, cadmium accumulates in soil more quickly than it is removed, resulting in a gradual increase in cadmium in soil and crops [37].

Due to the widespread and increasing nature of cadmium pollution, it is critical to identify methods to prevent and reduce exposures in early life. At a policy level, a number of interventions have aimed to reduce cadmium exposure through both remediation and prevention [38]. In the best-known example, hundreds of victims – primarily women – developed Itai-itai disease from cadmium pollution emanating from the Mitsui Kamioka mine in Toyama Prefecture, Japan, over several decades until the 1960s [39]. The victims eventually succeeded with extensive advocacy and legal action, and decontamination measures were implemented which significantly remediated the pollution [40]. Such extensive – and expensive – clean-up of cadmium pollution is exceedingly rare. In many cases, local and national governments in LMICs have called for restoring degraded environments [41] and (occasionally) stopping agricultural production in contaminated soils or watersheds [42]. The European Union set maximum levels for cadmium in foodstuffs in 2006 and decreased them in 2021 [43]. Targeted legislation, improved remediation, and more stringent controls through the 1998 Aarhus Protocol have contributed to significant progress in reducing cadmium emissions in high-income countries since 1990. Between 2005 and 2019, overall cadmium emissions in the European Union declined by 33% [44], although a few countries saw a small increase. The Convention on Long-Range Transboundary Air Pollution addresses cadmium emissions [12]. The WHO Framework Convention on Tobacco Control [45] aims to reduce tobacco smoke in indoor workplaces, public transport, and public places. While this convention could potentially reduce cadmium exposures, its success has been uneven and limited, especially in low-income countries, where smoking has increased [46].

Still, the United Nations Environment Programme notes that “these existing efforts are likely still inadequate to eliminate or minimise cadmium exposures from anthropogenic sources globally as a whole” [12]. Weak enforcement of quality control regulations and increased demand are, for example, important contributors to cadmium exposures from jewellery and toys in LMICs [24].

This review aims to identify and summarize the range of interventions implemented in LMICs for pregnancy, infancy, and childhood and assess their effectiveness for improving human health with the intention to inform policy development for reducing children’s exposure to toxic environmental chemicals.

## METHODS

### Theoretical model of how the interventions work

Environmental pollution from toxicants such as cadmium is increasing. Cadmium is a carcinogen and toxic to multiple organ systems, and early life is a vulnerable window for exposure and toxicity. Human exposures occur through food grown on contaminated land, air pollution, and unsafe consumer products. Due to rapid industrialization, LMICs are facing growing public health risks due to cadmium. Given the widespread nature of cadmium pollution, interventions have been tested to reduce early life exposures in a wide range of settings. Interventions typically take a prevention approach (preventing human exposure to cadmium), a remediation approach (cleaning up existing cadmium from the environment), or a treatment approach (medical or nutritional treatments that attempt to reduce the impact of cadmium in the body). Any of these approaches could in theory promote human health, but in practice, their success will depend on effectiveness, feasibility, cost, penetration, and social inequalities. Large-scale clean-up of existing cadmium pollution and reduction of future pollution will require identification of realistic and cost-effective, context-specific intervention strategies. Interventions that address early life exposures may be the most effective at reducing disease burden and promoting life-long health.

### Aims

This systematic review aimed to collect and assess research on programs, policies, and other interventions that aimed to reduce or prevent exposure to the toxic metal cadmium in pregnancy and childhood, specifically in LMICs. We assessed whether these interventions were effective at reducing or preventing cadmium exposures in early life. In addition, we assessed whether the interventions were associated with improved human health outcomes. Our methods were described in the protocol established before the review and registered with PROSPERO (CRD42021235435).

### Search strategy

The literature search was done on November 10, 2020, using the following databases: Scopus, Web of Science, PubMed, Global Health Medicus, and Greenfile. The search string was developed with guidance from a research librarian with expertise in systematic reviews. It used a combination of Medical Subject Headings (MeSH) and free-text keywords and was adapted by one reviewer to fit each database. Prior to conducting a full search, test searches were done to refine the search string with help from the second reviewer and the research librarian. Once the search strings had been developed, a full search was completed. The search was unrestricted by language and publication date. Reference lists of included articles were then hand-searched for additional relevant articles to screen. Search strings and numbers of results per database are provided in the **Online Supplementary Document** **(S1)**.

### Screening

Duplicates were removed first in Endnote (version X9.2) and then Rayyan [47]. Two double-blinded reviewers screened the titles and abstract for eligibility and labelled them with “include”, “exclude” or “maybe”. Any discrepancies were resolved by discussion between the two reviewers. After reaching consensus on eligible articles, the two reviewers performed double-blind full-text screening for inclusion based on the PICO criteria (**Table 1**). Full-text articles that were not found online were requested through the university library. If an article could not be found there either, it was excluded.

**Table 1:** Systematic review PICO criteria for inclusion

Population	Pregnant women, mothers, infants, children in LMICs; or products directly or indirectly used by these groups
Intervention	Policy, program, intervention, prevention, protection, mitigation, rehabilitation, counselling, clean-up, parenting program, health education; implemented to reduce human exposure to cadmium
Comparison	No intervention; “sham” intervention; or baseline
Outcomes	Human health outcome (eg, chemical body burden; body function including kidney, skeletal, respiratory, and nervous systems; and/or quality of life); and/or environmental contamination levels in areas relevant for children (homes, schools, etc.) and/or cost (if available)

To be included in this review, studies needed to be primary research that included an evaluation of cadmium chemical body burden in study participants (human biomonitoring) and/or in child- or pregnancy-relevant environments or products (environmental biomonitoring), within the context of an intervention. The populations of interest were children, infants, neonates, and pregnant women in LMICs. Interventions could include policies, programs, environmental clean-up/mitigation, rehabilitation, counselling, parenting programs, nutrition programs, clinical research, health education, or other relevant interventions at the household, community, or policy levels. Only primary research was included; secondary analyses such as reviews were excluded. Inclusion and exclusion of studies are reported based on PRISMA guidelines [48].

### Data extraction

Bibliography for all included articles is provided in the **Online Supplementary Document** **(S1)**. Included articles were extracted by one reviewer into a template tailored for this review; all extractions were checked by the second reviewer. The template included study characteristics, type of intervention, population studied, measurement of cadmium (either human or environmental biomonitoring), outcomes of interest, and comparators. For studies that did not report all data needed for data analysis, we requested the data from the corresponding author by email. Studies were grouped into either human or environmental interventions because the study methods and outcomes were categorically different and were better suited to different analysis approaches. The template used for data extraction is provided in the **Online Supplementary Document** **(S2)**.

### Quality assessment

Study quality and risk of bias were assessed using a template tailored to this review, based on modified GRADE criteria [49], in the categories of risk of bias, inconsistency, indirectness, imprecision, and publication bias. Included articles were all scored for quality by one reviewer; all scores were checked by a second reviewer. **Table 2** and **Table 3** display quality assessments for all included studies with heat colouring indicating quality (lower scores lighter, better scores darker). Quality of each included study was scored as no (0 points), partially (0.5), or yes (1 point) for each question (**Box 1**).

**Table 2:** Interventions for humans to reduce early life exposure to cadmium in LMICs*

Type of intervention	Reference	Description of intervention	Participants	n	Time to follow-up (approx.)	Human biomonitoring method	Colour code – human health outcome(s)	Quality score	Country
Nutritional supplements	Kelishadi et al., 2016 [50]	Daily provision of fresh jujube fruit (*Ziziphus jujuba Mill.*) compared to standard postpartum care, in a randomized design	Mothers postpartum	40	Eight weeks	Breastmilk at baseline and after intervention	Green – breastmilk metal concentrations after 2 mo of breastfeeding decreased in all participants. Consumption of jujube fruit was associated with a sharper decrease in cadmium level.	12.5	Iran
	Savchenko et al., 2014 [51]	Potential elimination of toxic elements using detoxal, a dietary supplement (enterosorbent) based on calcium alginate, (600 mg per day) for four weeks	Children, 5-7 y	42	36 d	Urine, hair and feces, before, during, and after treatment	Green – by the middle of the observation, children in the treatment group showed an increased concentration of heavy metals in the urine, as an indicator of elimination from the body. By the end of the fourth week, cadmium levels in urine were similar to those of the control group.	11	Russia
	Beletskaya et al., 2014 [52]	“Biocorrection” of imbalance of micronutrients and toxicants using pectin-based prophylaxis treatment to normalize microelement status	Children, 5-6 y	103	28 d	Blood, urine, hair	Green – pectin treatment was associated with improved cognitive performance in children, though there was no comparison group.	10	Ukraine
	Beletskaya et al., 2014 [52]	“Biocorrection” of imbalance of micronutrients and toxicants using pectin-based prophylaxis treatment to normalize microelement status	Pregnant women (second trimester)	89	21 d	Blood, urine	Green – cadmium level in blood decreased 14.3% while increasing concentration in urine.	10	Ukraine
	Bisanz et al., 2014 [53]	Provision of locally produced probiotic yogurt containing *Lactobacillus rhamnosus* and supplemented with Moringa, a micronutrient-rich plant	Children, 6-10 y	36	25 d	Blood samples at baseline and after intervention	Blue – non-significant differences between intervention and control groups.	10	Tanzania
	Bisanz et al., 2014 [53]	Provision of locally produced probiotic yogurt containing *Lactobacillus rhamnosus* and supplemented with Moringa, a micronutrient-rich plant	Pregnant women	37	102 d	Blood samples at baseline and after intervention	Blue – non-significant differences between intervention and control groups.	10	Tanzania
	El-Soud et al., 2011 [54]	Provision of a diet regimen high in fruits, vegetables, milk and protein and low in calories. Health education and support were given to children and their mothers to insure following the dietary program.	Children 11-14 y from primarily low-middle social and economic classes	65	Two months	Urinary cadmium at baseline and after intervention	Green – significant reduction was observed in cadmium. Mean urinary cadmium concentration decreased from 12.8 to 9.4 µg per liter following intervention, though there was no comparison group.	9	Egypt
Rice cooking method	Shafiei et al., 2017 [55]	Educational program on frequency of rice consumption and manner of cooking (kateh vs pilaw). Control group was given no consultation. Health Belief Model intervention group received consultation with researchers. Ecological intervention group had a larger group of participants including researchers, family members, friends, and colleagues, with use of social media, training sessions, and telephone consultation.	Women, 18-50 y	240	Six months	No	Green – both intervention groups showed an increase in consumption of local rice (described as uncontaminated) and decrease in consumption of foreign rice. The Ecological model group showed increase in pilaw cooking method and stronger social support compared to the other groups. The Ecological model group participants tended to be higher educated and have more relevant knowledge prior to the intervention.	10	Iran
Medicine and/or clinical care	Luzhetsky et al., 2018 [56]	Treatment with elimination, membrane stabilizing, antioxidant and nootropic technologies, physiotherapy (ultrasound therapy, inductothermy), and exercise. 21-d courses repeated twice over one year.	Children, 4-15 y with physical developmental disorders and nutritional deficiency	62	12 mo	Blood, urine	Green – treatment group blood cadmium level significantly reduced from 0.0004 to 0.0001 while reference group did not significantly change.	10.5	Russia
	Blaurock-Busch et al., 2012 [57]	Nutritional supplementation and DMSA oral chelation	Children with autism, 3-9 y (37 boys, 7 girls)	44	Six months of chelation concluded with follow-up CARS evaluation	Urinary cadmium at baseline and after intervention	Green – following six months of chelation and nutritional intervention, there was a significant increase in cadmium excreted from baseline to follow-up. There was a significant positive correlation between baseline urine cadmium with taste, smell, and touch responses. No comparison group.	9.5	Saudi Arabia

LMIC – low- and middle-income country

*Human health outcome(s) colour coding explained: green for significant potential to improve human health, yellow for inconclusive/mixed effect, blue for neutral effect, black for detrimental effect.

**Table 3:** Environmental remediation interventions to reduce early life exposure to cadmium in low- and middle-income countries*

Type of intervention	Reference	Description of intervention	Biomonitoring method	Measure of human exposure (direct or indirect)	Cost†	Colour code - potential for human health impact	Data quality score	Country
Agricultural soil remediation	Nawab et al., 2018 [58]	Organic amendments (biochar, farmyard manure, and peat moss) were used at different application rates (1%, 2% and 5%) in mining-impacted agricultural soil to immobilize toxic metals	Cadmium was measured in the pea and chili plants	Human exposure was measured by daily intake of metal which is based on concentration of metal in vegetable, daily intake of vegetables, and body mass for adults (73 kg) or children (32.7 kg). Hazard quotient index was based on daily intake and reference dose.	-	Green – compared to control, all the tested organic amendments, at application rates of 1%, 2% and 5%, decreased the bioavailability of toxic metals concentrations in soil, their bioaccumulation in pea and chili, and corresponding daily intake and health risk index. Biochar soil application demonstrated the highest reduction in daily intake of metal (approx. 20%-35% reduction for children and adults), compared to farmyard manure or peat moss, for all plants studied.	13	Pakistan
	Khan et al., 2014 [59]	Sewage sludge biochar was amended into contaminated rice paddy soil (rates of 5% and 10%) at a controlled greenhouse in an Mn-Zn mining area. After 15 d, two uniform seedlings were transferred into flooded experimental pots containing the contaminated soil or biochar treated soil.	Cadmium was measured in soil and in rice plants after harvesting	Estimated daily intake based on exposure duration (70 y), exposure frequency (365 d per year), concentration in rice, average body weight, life expectancy, and rice intake rate (398.3 g/d for adults). Hazard quotient was based on estimated daily intake and oral reference dose.	Production costs: US$0.08-0.59 per kg. Transportation and other costs not included.	Green – sewage sludge biochar addition to Mn–Zn mine impacted soil was effective in suppressing bioaccumulation of cadmium in rice plants and consequent reduction in estimated daily intake. At high application rates (10%), the hazard quotient for cadmium was reduced below acceptable limits.	13	China
	Khan et al., 2018 [60]	Four organic amendments (maize comb waste, rice husk, hard-wood derived biochar, bagasse) were added to tomato and cucumber plants in mine degraded soils to investigate availability, uptake, and translocation of cadmium.	Concentration of cadmium in the amended plant was divided by the cadmium concentration in the control plant to determine concentration index (CI)	Daily intake of cadmium based on concentration of cadmium, daily intake of vegetables, and bodyweight. Health risk index and target hazard quotient also derived.	-	Yellow – results varied. Biochar was an effective amendment for reducing cadmium plant uptake and health risk index via dietary exposure and performed best among the four amendments. The use of untreated plant residues (maize comb waste) was not effective in the immobilization of cadmium and rather they enhanced the uptake of cadmium.	11.5	Pakistan
	Taghipour et al., 2020 [61]	Organic waste (rice husks and straw) was added to potted tomato plant soil to investigate the potential reduction in uptake of toxic metals from industrial solid wastes from nearby ceramic, sugar, and stone cutting factories.	Cadmium was measured in the soil samples and the tomato plant after growth. Uptake was measured as a ratio of plant to soil concentration.	Human exposure was measured through an EDI (estimated daily intake) for tomato based on average body weight for adults and children. Health risk index was calculated by dividing EDI by reference dose of cadmium.	-	Green – application of organic wastes (especially rice husks) significantly reduced the mobile fraction of toxic metals in both types of soil and in different parts of tomato plants and lowered the HRI.	10.5	Iran
	Wang et al., 2019 [62]	Application of biochar, phosphate materials and compost on contaminated soils to immobilize cadmium and lead	Cadmium uptake measured in 20 types of local leaf vegetable cultivars. Cadmium content tested after 2 y of growing with 3 types of soil remediation	Hazard quotient calculated for children and adult females, based on exposure frequency, duration (years), and heavy metal concentration in the edible parts of the vegetable.	US$3885 per ha for raw materials of B3P3C2 treatment	Green – before intervention, all hazard quotients were above 1 indicating serious risk level. Remediation decreased all hazard quotient values by at least 66%. Biochar was the most effective treatment to reduce plant uptake of cadmium. However, high-cadmium accumulating cultivars of vegetables should be avoided due to the health risks even after remediation.	9.5	China
	da Silva et al., 2017 [63]	Corn (*Zea mays*) and castor bean (Ricinus communis) were used for phytoextraction induced by chelating agents or phytostabilization	cadmium uptake was measured by coupled plasma optical emission spectrometry as the net removal from the soil to the shoots	Hazard index for children age 1-6 y for non-carcinogenic risk based on daily exposure dose and reference dose.	-	Yellow – the mean estimated time for remediation of the area using phytoextraction was high, ranging from 76 to 259 y, and therefore this technique is not a viable method for decontaminating soils in the study area.	9	Brazil
Indoor air filter	Barn et al., 2018 [64]	Pregnant women in the intervention group received one or two portable HEPA filter air cleaners depending on the size of their apartment, and air cleaners were used from the first home visit until childbirth. For smaller apartments (<40m2), air cleaners were placed in the main living area of the home, and for larger apartments (≥40 m2) the second unit was placed in the participant's bedroom.	Yes, at baseline (5-18 weeks gestation) and late (24-37 weeks gestation): blood (n = 382) and hair (n = 125) samples; air pollution measured during home visits	Samples from pregnant women (≤18 weeks); n = 511 at follow-up (initially 540 randomized)	US$200-300	Green – in this randomized controlled trial, air cleaners substantially reduced indoor PM2.5 concentrations and secondhand smoke exposures as measured by blood cadmium among a group of pregnant women in a highly polluted city.	14.5	Mongolia
	Brehmer et al., 2020 [65]	Atmosphere® air purifier (Amway, USA) with HEPA filter was placed in the participant's bedroom for a 2-week period with real and false filtration scenarios. Study investigated oxidative potential of PM2.5.	Cadmium air measurements for 43 children ages 5-14 with physician diagnosed asthma	Cadmium was measured in the home at breathing height, outdoor on balcony or window, and in a sampling backpack carried by the children at baseline and after intervention.	US$100-500 per air cleaner, US$50-100 per filter (needs to be changed every 6 mo)	Yellow – cadmium showed variable results after filtration. Indoor cadmium levels higher with real filter; lower with real filter for outdoor and personal exposure. Indoor air cleaner reduced the measured oxidative potential of personal exposure to PM2.5.	12.5	China
	Brehmer et al., 2019 [66]	Atmosphere® air purifier (Amway, USA) with HEPA filter was placed in the participant's bedroom for a 2-week period with real and false filtration scenarios. Study investigated chemical composition and pollution sources.	Cadmium air measurements for n = 43 children ages 5-14 y with physician diagnosed asthma	Cadmium was measured in the home at breathing height, outdoor on balcony or window, and in a sampling backpack carried by the children at baseline and after intervention.	US$100-500 per air cleaner, US$50-100 per filter (needs to be changed every six months)	Green – indoorair cleaner was effective at reducing indoor concentrations of PM2.5-bound metals and reducing personal exposure to some potentially hazardous elements (eg, As, Cd, Sb, Pb) by reducing exposure to contributions from some outdoor sources that had migrated indoors. However other pollution sources outside the bedroom were not reduced by the air cleaner.	12	China
Cookware and microwaving	Weidenhamer et al., 2017 [67]	Four aluminium pots which leached arsenic, cadmium and/or lead in initial testing, were coated with a fluoropolymer finish (Xylan), cured, and evaluated for cadmium leaching.	Cadmium was measured in 250 mg water boiled in pot	No	US$10 000-20 000 for spraying equipment. Xylan coating material cost is US$1.00 for 3 pots.	Yellow – the results from the fluoropolymer treatment (Xylan) of artisanal aluminium cookware suggest that coating aluminium cookware may be effective at substantially reducing the hazardous levels of metals observed leaching during typical cooking. However, Xylan is a perfluorinated compound (PFAS) which is linked to worse health.	10.5	Bangladesh, Guatemala, India, Indonesia, Ivory Coast, Kenya, Nepal, the Philippines, Tanzania, and Vietnam
	Wang et al., 2014 [68]	Microwaving food (carrot, potato, lotus root, white radish, sweet corn, long grain rice, soybean, fleshy prawn, eastern oyster, kelp, common carp) to determine if microwave cooking reduces the concentration of bioaccessible cadmium.	Microwaved food samples were processed through laboratory digestion and evaluated for uptake in three simulated digestive uptake phases	Daily exposure calculation using the ingestion rate for the Chinese population and cadmium concentration.	-	Yellow – effect of microwave cooking upon the total cadmium and cadmium bioaccessibility varied by type of food matrix. Microwave cooking did not significantly change cadmium concentration in most investigated foods except in potato (decreased cadmium), lotus root, and eastern oyster (increased cadmium). Most microwaved food samples showed lower cadmium bioaccessibility than unprocessed counterparts (up to 68% lower), indicating that this process could modulate less absorption of cadmium.	10.5	China
Rice preparation (laboratory)	Shariatifar et al., 2020 [69]	Various pre-cooking (washing, soaking) and cooking processes (traditional and rinse) of rice on the amount of toxic and essential elements in the various brands of rice	Cadmium was measured in 144 samples of rice after different cooking methods using laboratory methods	For children and adults, hazard quotient was calculated using the reference dose, and estimated daily intake was based om metal concentration, ingestion rate, body mass, exposure frequency, and mean time.	-	Green – it was concluded that the best method for removing the highest amount of toxic metals while maintaining high levels of essential elements was the rinse method (washing rice 5 times followed by soaking for 5 h). The findings of health risk assessment exhibited that the THQ values for the 95th percentile consumer were not at considerable non-carcinogenic risk	12.5	Iran
	Zhuang et al., 2016 [70]	Contamination level of cadmium in three different types of rice was assessed at low, medium and high concentrations. The concentration was assessed in cooked and raw rice, and at three different water:rice cooking ratios (2:1, 4:1, 6:1) to see if there is a reduction in concentration when utilizing more water.	Cadmium concentration was assessed in rice using laboratory methods and digestion of rice samples	Estimated daily intake and target hazard quotient	-	Yellow – application of a low volume of water during cooking to dryness of rice was able to remove total cadmium by about 10% for some types of rice. Use of medium or high volumes of water did not show any effect on cadmium bioaccessibility. Daily intake of cadmium from rice B by adults and children exceeded toxicological reference values.	12.5	China
	Naseri et al., 2014 [71]	Assess the effect on the cadmium content in the rice post cooking with two methods: kateh cooking (all water evaporated; steaming) and pilaw cooking (water drained after cooking).	3 rice brand samples were cooked, digested, and analyzed for cadmium concentration	Estimated daily intake calculation using cadmium concentration, amount of rice consumed daily, and body weight.	-	Green – toxic metal contents in rice grains and estimated cadmium intake were reduced following both kateh (all water evaporated) and pilaw cooking (water drained after cooking), with pilaw typically showing larger reductions in cadmium levels.	10	Iran
Environmental policy	Díaz-Barriga et al., 1997 [72]	Lead smelting (which emitted lead, cadmium, and arsenic) on the US side of the border was halted in August 1985	Soil and dust measurements	Cadmium was measured in soil at places frequented by children and in dust from windows of participating children	-	Yellow – soil cadmium levels were approximately 2 to 3 times higher within a 1.8 km radius of the former smelter than to the control site 25 km away. Cadmium levels in dust did not differ at the three sampling sites within the 1.8 km radius of the former smelter.	11.5	Mexico
	Cao et al., 2017 [73]	Multiple control measures were implemented in a mining area: dam wall was constructed to trap sediments and waste water from the Dabaoshan mine into the Hengshishui River and downstream areas; all small scale and illegal mining and refining activities were forbidden; devices for waste decontamination and greening reconstruction were implemented; and discharge of wastewater and agricultural pollution were restricted.	Cadmium was measured from water samples from 7 different sampling sites along the river basin, 3-8 samples per site.	Human exposure was measured through a hazard quotient (HQ) which was found by dividing average daily dose (ingestion and dermal contact) with the reference concentration. This was evaluated for children (age 0-5 and 6-17) and adults (>18).	-	Yellow – close to the mining area in the Beijiang River basin, heavy metal pollution remained; strict pollution control measures are still called for to reduce health risks and improve drinking water quality. Farther from the mining area, soluble metal concentrations in the river basin were lower than the previous contamination levels prior to implementation of these strict measures and were lower than the thresholds regulated in Chinese national water quality standards. Hazard index for metalloid exposure via water was highest for children 0-5 y.	13	China
	Jin et al., 2020 [41]	Management measures published by the Guangxi Zhuang Autonomous Region in 1992 requiring mines in the region to implement environmental protection measures regarding their emissions of waste water, waste gas, and solid waste; reclaim land destroyed by mining; and, later, to improve polluted rivers.	Cadmium plasma levels 13 and 25 y after initial policies enacted	382 women, 18-30 y, healthy, non-pregnant, non-lactating, non-occupational exposures	-	Green – the median plasma concentrations (P25-P75) decreased by 0.05 ng/mL from 2005 to 2012. Significantly lower ORs for cadmium were found for greater egg consumption (>1 × day), more fresh fruit consumption (>3 × week), and location Ganxu (vs Taiping). Proportion of women who had cadmium level over the upper reference limit decreased from 92.0% to 82.4%. Cadmium was not associated with vegetable consumption. Seafood was not consumed much in this population, and almost no women were exposed to smoking.	11.5	China
	Jan et al., 2010 [74]	Wastewater stream originating in industrial zone located in Hayatabad was used for irrigation due to freshwater shortage. Soil samples from a contaminated agricultural area and from less-contaminated areas were compared.	Concentration of metals in each type of soil were compared to derive metal transfer factor	Health risk index based on daily metal intake and oral reference dose.	-	Yellow – vegetables grown in the contaminated soil showed elevated levels of metal; however, soil cadmium concentrations were found within WHO/FAO limits. Control vegetables showed lower levels of metals than those grown in contaminated soil.	9.5	Pakistan

LMIC – low- and middle-income country

*Potential for human health impact colour coding explained: green for significant potential to improve human health, yellow for inconclusive/mixed effect, blue for neutral effect, black for detrimental effect.

†Cost information given where available.

Box 1 **Modified GRADE criteria [49] used to assess study quality.**

1. Is the study design a randomized trial?
2. Does the analysis include assessment of dose-response effects? (i.e., dose/magnitude of intervention linked to magnitude of health response/effect)
3. Did the analysis include at least 3 of the most relevant confounders?
4. Was there blinding of participants and personnel (i.e., no potential for performance bias)?
5. Was an objective outcome used?
6. Less than 20% participant dropout from enrollment to analysis? (i.e., no potential attrition bias)
7. Were all relevant data reported for the outcome of interest (i.e., no potential selective reporting)? (no potential reporting bias)
8. No other biases reported? (i.e., no potential of other bias)
9. Did the intervention end as scheduled (i.e., not stopped early)?
10. Do the authors provide confidence intervals for effect sizes?
11. Was the direction of effect consistent across participants?
12. Was the included outcome a direct outcome (i.e. not a surrogate/proxy outcome)?
13. Was the outcome timeframe sufficient?
14. Were the conclusions based on direct comparisons?
15. Study included at least 10 participants in each group?
16. Was there no evidence of serious harm associated with treatment?
17. There was no industry influence on the study? (i.e., author affiliations or funding sources)
  Scoring: No = 0 points
  Partially = 0.5 point
  Yes = 1 point

### Data synthesis and analysis

Synthesis without meta-analysis (SWiM, also known as narrative synthesis) [75] was used for analysis of the data extracted through the systematic review. The SWiM approach was adopted due to the wide heterogeneity of study designs and materials used in the reviewed studies (eg, diverse cadmium sources; human body burden vs environmental biomonitoring; wide variety in study quality). SWiM allowed for the best use of the available data and for capturing the complexity for this research question.

Because few studies reported data suitable for meta-analysis and few were of high quality, the review authors opted to present relevant information in table format using the units provided by the original studies. There was a lack of similar-enough data to calculate standardized effect sizes or create a standardized metric for this review.

Articles were categorized as either human interventions (**Table 2**) or environmental interventions (**Table 3**) and analysed separately due to large methodological differences. In analysing the findings, reviewers considered both the study’s quantitative results the study authors’ overall interpretation of their own intervention’s success and limitations. Reviewers assessed these for direction of effect, so the synthesis primarily used a vote counting approach based on the reported direction of effects. For each study, the intervention was categorized in **Table 2** and **Table 3** based on our assessment of the reported results and study authors’ interpretations as

- Green: positive. This means the results indicated significant potential to improve human health and that all reported findings and author interpretations pointed in the same direction towards better health.
- Yellow: inconclusive/mixed. This means that some of the results indicated a potential for better health outcomes while other results were either neutral or suggested a potential for worse health;
- Blue: neutral. This means that the results indicated neither the potential for better nor worse health; or
- Black: detrimental. This means that the reported findings indicate that the intervention was associated with worse health outcomes.

This review explored heterogeneity in the reported effects using tables and visual elements. Extracted data on the following factors was presented and compared: intervention characteristics, participant age (for human biomonitoring studies), time to follow-up, biomonitoring methods, cost, study quality, and human health outcome (for human biomonitoring studies) or estimated human health impact (for environmental studies). Tables are organized by intervention type. For human studies, results are presented separately for children and women, as these were two distinct target populations for interventions. **Table 2** and **Table 3** include information on all studies, regardless of study quality score. However, we emphasize the findings with highest study quality scores: within each sub-category of intervention from the tables (eg, human intervention to indoor air filter), results from the studies with highest quality are also described briefly in the text.

## RESULTS

**Figure 2:**
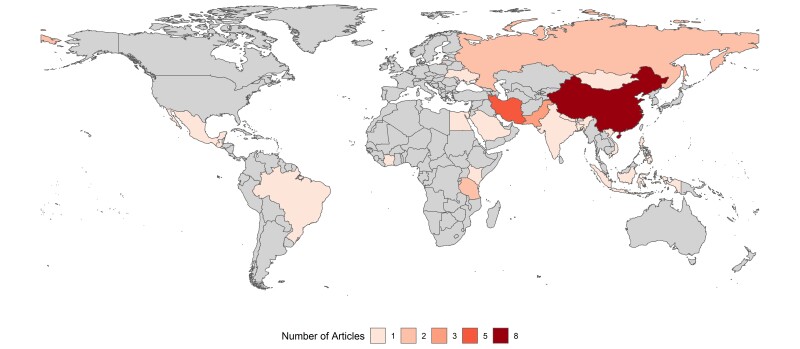
Map indicating study locations for included studies. China and Iran were the countries with most included studies.

**Figure 3:**
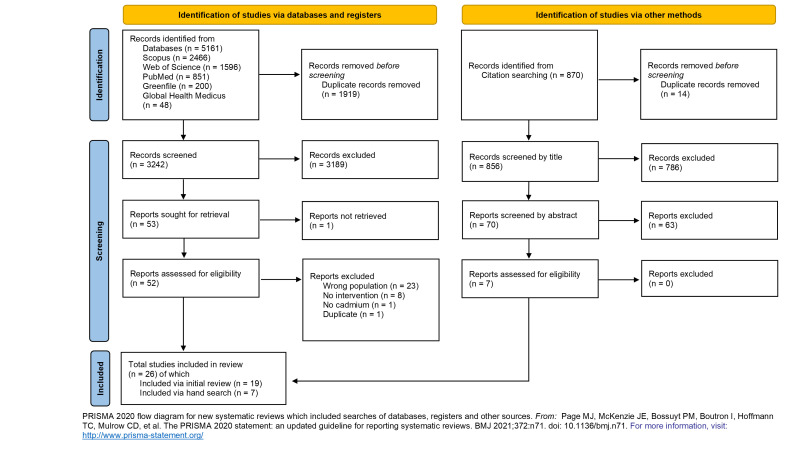
PRISMA flowchart for inclusion in systematic review.

We systematically reviewed five databases (Scopus, Web of Science, Global Health Medicus, Greenfile, and PubMed) and found 4098 articles, 26 of which were relevant for this review (**Figure 2** and **Figure 3**). The review found a mix of studies of interventions for humans and for environmental remediation, four of which examined policy-level changes [41,72-74].

### Human interventions

Studies of interventions for humans (**Table 2**) focused on nutritional supplements (n = 7), medicine/clinical care (n = 2), or rice cooking education (n = 1). These studies generally involved human biomonitoring (eg, urine, blood, breastmilk, hair, or faeces) in children, pregnant or lactating women, or women of childbearing age. Shafiei et al. [65] did not include biomonitoring, but assessed change in diet. Most studies recruited a typical community population; a few examined subgroups, namely children with physician-diagnosed asthma developmental disorders and nutritional deficiency [56], autism [57], or children from middle and lower socioeconomic status [54]. Notably, two studies [52,53] evaluated interventions on both children and pregnant women, and the results are therefore listed on separate rows in **Table 2**. Indoor air cleaners demonstrated effectiveness at reducing indoor concentrations of cadmium when deployed for two trimesters during pregnancy [64], and showed mixed effectiveness when deployed over a shorter period (two weeks) during childhood [65,66]. Nutritional and medical interventions ranged from provision of fresh jujube fruit (*Ziziphus jujuba Mill.*) [50] or probiotic yogurt (containing *Lactobacillus rhamnosus*) during pregnancy/postpartum [53], to chelation therapy in children [57]; studies were too small and dissimilar to compare here and warrant more extensive clinical investigation.

### Environmental interventions

Most studies investigated environmental remediation interventions (**Table 3**), including for cooking. The most common was agricultural soil remediation (n = 6), followed by environmental regulations (n = 4), indoor air filtration (n = 3), rice cooking method (n = 3), and cookware and microwaving (n = 2). In general, these studies used environmental biomonitoring and indirect measures for estimating human health impact (eg, estimated daily intake, hazard quotient); Barn et al. [58] used human biomonitoring, but is included in **Table 3** with other air filtration studies for consistency. Remediation of agricultural soil using biochar reduced the estimated children’s daily intake of cadmium ; application of rice husk waste to soil also lowered the child health risk index linked to tomato consumption [61]. Other types of organic waste were less effective or ineffective at immobilizing cadmium and (in one case) even enhanced uptake of cadmium into food plants [60]. Rice cooking in either high or low water did not show conclusive results for reducing cadmium content [70], but pre-rinsing five times and soaking of rice was associated in one study with lower hazard quotient for children and adults [69].

### Location

We reviewed only studies from LMICs. The 26 studies were conducted in 21 different countries (**Figure 2**), with one study [67] accounting for 10 of those. China (n = 8), Iran (n = 5), Pakistan (n = 3), and Russia (n = 2) were the only countries where more than one study had been conducted, with studies in China and Iran accounting for 50% of the studies.

### Effectiveness

Of the 26 studies, 14 showed significant potential to improve human health, nine showed inconclusive/mixed effect, and three showed neutral effect. The majority (n = 7) of the human intervention studies indicated associations between the intervention and improved health, while two studies showed a neutral effect. From the environmental studies, seven showed that the intervention improved health, but most environmental interventions (n = 9) showed an inconclusive/mixed effect, while one demonstrated a neutral effect on health outcomes. No studies indicated only detrimental effects of the intervention.

### Intervention cost

Six studies provided cost information for interventions, and one estimated financial benefit of the intervention. Of these studies, HEPA and activated carbon air filter (n = 3) and soil amendment (n = 2) had more than one. The cost of one HEPA and activated carbon air filter was in the range of US$100-500, with a replaceable filter which costs US$50-100 that must changed every six months. The cost of soil amendment depended on the materials used, with one study [59] reporting US$0.08-0.69 per kilogram of sewage sludge biochar produced and another [62] reporting US$3885 per hectare for biochar, phosphate materials and compost raw materials. Cost information was also retrieved from Weidenhammer et al. [67] where the cost for Xylan® coating of cookware was found to be US$1.00 for three pots, with a cost of equipment ranging from US$10 000 to US$20 000. Only the study by Luzhetsky et al. [56] estimated a financial benefit of the intervention, reporting prevented annual GDP losses of 13246 rubles per person (approximately one week of average 2018 wages) with technologies for treating physical development disorders.

### Other pollutants

Of the 26 studies included, 23 investigated other metals or pollutants in addition to cadmium. The most common contaminants included were lead (n = 19), arsenic (n = 14), zinc (n = 10), chromium (n = 9), copper (n = 8), nickel (n = 7), and manganese (n = 7). Additionally, iron, aluminum, cobalt, mercury, antimony, vanadium, molybdenum, and titanium were reported in more than one study. Outside of metals, particulate matter smaller than 2.5 µm (PM_2.5_) were assessed in two studies focused primarily on air pollution.

### Study quality

The average study quality for all studies included was 11 (scale of 0 to 17 best), based on a modified GRADE scale [49] (**Box 1**). Two of the human intervention studies were of average quality, while seven were below average; ten of the environmental studies were of average quality, while seven were below average. Five studies randomized the participants to treatment or control group [50,53,55,64,65], five reported a dose-response effect [58,59,62,64,70], and only three studies provided confidence intervals for effect sizes [50,64,73]. Three studies blinded the personnel and participants [65,66,69]. Overall, Barn et al. [64] was rated as the study with highest overall quality, with a score of 14.5. Data used in study quality assessment is provided in the **Online Supplementary Document** **(S3)**.

### Robustness of the synthesis

These studies included substantial heterogeneity, which provided limited evidence behind any single type of intervention. Where possible, similar data points were identified and extracted from all studies and presented in a transparent way via tables and text. This review attempted to reduce bias by using a modified GRADE quality scale. Given the widespread nature of cadmium contamination, interventions targeting different exposure pathways (such as food vs air pollution) will by nature have different expected levels of effectiveness, which also make them difficult to compare. Moreover, social inequalities in health (such as access to nutritional foods, health care, and education) will impact the context surrounding each intervention and are beyond the scope of this preliminary review. Nevertheless, taken together, these interventions studies underline the challenges of addressing cadmium as a public health problem in early life, and the limited tools available to solve it.

## DISCUSSION

This systematic review identified a range of interventions aimed at reducing early life exposures to cadmium in LMICs. A total of 26 studies from 21 countries were included, targeting children, pregnant and postpartum women, and women of child-bearing age. All in all, these studies provided some evidence of effectiveness, but none appeared to offer a realistic solution for cadmium pollution at scale. Agricultural and food preparation interventions were relatively frequent, particularly related to rice, though deploying the agricultural interventions at scale would require substantial initial investment. Air filtration studies indicated some effectiveness in reducing indoor cadmium exposures during pregnancy. Four studies used long-term follow-up design to examine children’s cadmium exposure risks related to smelting, mining, and industrial pollution [41,72-74], none of which eliminated the cadmium pollution. The knowledge gaps identified by this review can be addressed by higher-quality studies and innovative interventions. Except for one educational program [55], all studies took a remediation approach, which may have contributed to the overall limited effectiveness.

Children are more exposed to cadmium than the general population, as are vegetarians, smokers, and people living in highly contaminated communities [76]. Cadmium has drawn comparisons to lead in terms of widespread exposures and multi-system toxicity. Like lead, pesticides, and other environmental toxicants, cadmium has many entry points into the human exposome during pregnancy, infancy, and childhood. Like for other environmental toxicants, efforts to mitigate children’s exposures to cadmium have failed to sufficiently protect children’s health.

In this review, intervention designs ranged widely in their attempt to reduce human exposure to cadmium. This underlines the need to prevent uptake into the human body directly (eg, remove cadmium from foods at home, outcompete nutritionally, filter indoor air) and indirectly (eg, close smelters, remove cadmium from agricultural soils). Prevention of new exposures to environmental toxicants must be prioritized along with large-scale investment in remediation, especially in the Global South. At the same time, it is important to avoid “regrettable remediation” whereby the remediation efforts cause more damage than the substance itself [77]. Intervention research is useful for understanding the cost-benefit trade-offs and, in the case of environmental pollution, helping guide evidence-driven, effective allocation of limited resources to protect health.

LMICs face substantially different pollution mixtures than HICs – such as worse air pollution, garbage burning, and fewer regulations on chemicals in consumer products. What’s more, the chemical cocktails children are exposed to likely exert larger toxic effects since they mix with other inequities that affect health and brain development, such as poverty, low-quality housing, malnutrition, and social stressors. Twenty-three of the studies reviewed here collected data on multiple metals or other pollutants, most commonly lead (n = 19), arsenic (n = 14), zinc (n = 10). However, none reported results from mixture analyses with cadmium. Yet the toxicity of the neurotoxicant lead appears to increase in the presence of high levels of other metals such as cadmium [5]. Many environmental interventions target routes of exposure, rather than single toxicants. A better understanding of how interventions affect exposure to chemical mixtures – and their potentially synergistic effects – would therefore be valuable and better tailored to children’s real-world exposures.

Foodborne cadmium was estimated to account for 12 224 illnesses, 2064 deaths, and 70 513 DALYs in 2015 [78], making it a significant contributor to global health hazards. Rice, a staple crop for billions around the world, is a source of exposure to toxic metals. Extensive research has examined how health risks from rice are influenced by different rinsing, soaking, and cooking methods (eg, high water, low water, high-pressure cooking, and microwave) [79]. While the studies reviewed here did show some evidence for removal of cadmium risks related to specific types of rice preparation, types of rice and study methods differed. Cadmium-related risks from rice consumption should be a focus of additional research and action to remediate contaminated rice paddies. Additional studies have explored alternate cooking methods (eg, parboiling husked rice) [80,81] and potential agricultural interventions [19], but did not include human exposure estimates and were therefore not included in this review.

In Iran, cadmium contamination has been identified in staple foods including cereal, legumes, canned tuna fish, vegetables, fruit juice, and egg; approximately 75% of rice samples had cadmium levels higher than the national maximum value (0.06 mg/kg) [82]. Between 2013 [83] and 2016 [50], cadmium and lead levels in human breastmilk increased in Isfahan, a large industrial city in Iran, indicating increasing pollution.

A meta-analysis of 343 studies reported lower concentrations of cadmium in organic foods, particularly cereals (eg, wheat), compared to conventional crops [84]. While a full assessment of organic farming practices was beyond the scope of this review, six studies did investigate soil remediation interventions. Use of biochar reduced estimated children’s daily intake of cadmium. The environmental impact of burning plant waste to produce biochar was not taken into consideration in these studies, however, and may complicate the picture.

Certain probiotics may offer an opportunity to prevent uptake of metals such as cadmium into cells in the digestive tract; however research into such technologies is still in its infancy [85]. Bisanz et al [53] did not find evidence for a protective effect in Tanzania of provision of locally produced probiotic yogurt containing *Lactobacillus rhamnosus*, either during pregnancy or childhood.

Only one included study took a prevention approach. Shafiei et al. [55] tested an educational intervention on knowledge of rice contamination. While the characteristics of the three sample groups were not well-balanced, both intervention groups showed an increase in consumption of local rice (described as uncontaminated) and decrease in consumption of foreign rice. Health education and parenting programs have been examined for other toxicants such as lead [86], bisphenol A [87], and various consumer materials containing hazardous chemicals [88], with mixed results [89]. Education around children’s environmental health is an important area for additional prevention efforts and study.

Currently, no effective means for reducing cadmium absorption following inhalation have been reported, and no treatments other than supportive care and avoidance of additional risk are presently known for reducing latent effects on lung function [2]. Chelation has been studied as an intervention for children with known exposure to cadmium and specific diagnoses, such as autism [57]. Research on chelation treatment following cadmium exposure is continuing [90], but currently, some forms of chelation may be useful while others are ineffective, likely due to the rapid uptake of cadmium into tissue [2]. Some chelators may even worsen cadmium toxicity. Prevention is always considered better than treatment.

### Limitations

Tobacco use is therefore a large source of exposure globally; for heavy smokers, daily intake of cadmium from smoking may exceed that from food. Due to extensive research on smoking and tobacco cessation (eg, [46]), this study did not focus on smoking-related cadmium exposures. In general, the included studies showed moderate to low quality due in several cases to lack of comparison groups, non-randomized designs, little detail provided to support results (eg, no confidence intervals reported), small sample sizes, and short follow-up durations. This review relies on intervention effectiveness reported by each of the studies included. For these reasons, in addition to the wide variety of interventions studied, meta-analysis was not possible, and effect sizes are therefore not standardized or possible to compare directly here. While this reduces this study’s ability to synthetize the effectiveness of any one specific type of analysis, it does provide a more comprehensive summary of the types of intervention research that have been implemented and the limitations in the existing evidence base.

## CONCLUSIONS

Cadmium remains a threat to human health worldwide. Children living in rapidly industrializing LMICs face additional risks for exposure to cadmium and countless other environmental toxicants [91]. Costs of cadmium remediation will likely require extensive investment in the short term, given the costs associated with clean-up interventions. Prevention of exposure in early life remains a stronger approach than remediation, and they must be prioritized together to promote lifelong health and well-being.

## Additional material:


Online Supplementary Document

## References

[1] IARC. Cadmium and cadmium compounds. Arsenic, Metals, Fibres, and Dusts. Lyon, France; 2012. Available: https://publications.iarc.fr/Book-And-Report-Series/Iarc-Monographs-On-The-Identification-Of-Carcinogenic-Hazards-To-Humans/Arsenic-Metals-Fibres-And-Dusts-2012

[2] ATSDR. Toxicological Profile for Cadmium. 2012 Sep p. 487. Available: https://www.atsdr.cdc.gov/ToxProfiles/tp5.pdf

[3] Liu Z Cai L Liu Y Chen W Wang Q Association between prenatal cadmium exposure and cognitive development of offspring: A systematic review. Environ Pollut. 2019;254:113081. . 10.1016/j.envpol.2019.113081 31473391

[4] Nogawa K Tsuritani I Kido T Honda R Yamada Y Ishizaki M Mechanism for bone disease found in inhabitants environmentally exposed to cadmium: decreased serum 1α, 25-dihydroxyvitamin D level. Int Arch Occup Environ Health. 1987;59:21-30. . 10.1007/BF00377675 3793241

[5] von Stackelberg K Guzy E Chu T Henn BC Exposure to Mixtures of Metals and Neurodevelopmental Outcomes: A Review. Risk Anal. 2015;35:971-1016. . 10.1111/risa.12425 26096925 PMC5108657

[6] Röllin HB Kootbodien T Channa K Odland JØ Prenatal Exposure to Cadmium, Placental Permeability and Birth Outcomes in Coastal Populations of South Africa. PLoS One. 2015;10:e0142455. . 10.1371/journal.pone.0142455 26544567 PMC4636426

[7] Sripada K “Beginning with the Smallest Intake”: Children’s Brain Development and the Role of Neuroscience in Global Environmental Health. Neuron. 2017;95:1242-5. . 10.1016/j.neuron.2017.08.009 28910615

[8] Xu S Hansen S Sripada K Aarsland T Horvat M Mazej D Maternal Blood Levels of Toxic and Essential Elements and Birth Outcomes in Argentina: The EMASAR Study. Int J Environ Res Public Health. 2022;19:3643. . 10.3390/ijerph19063643 35329330 PMC8954125

[9] Skogheim TS Weyde KVF Engel SM Aase H Surén P Øie MG Metal and essential element concentrations during pregnancy and associations with autism spectrum disorder and attention-deficit/hyperactivity disorder in children. Environ Int. 2021;152:106468. . 10.1016/j.envint.2021.106468 33765546

[10] Gustin K Tofail F Vahter M Kippler M Cadmium exposure and cognitive abilities and behavior at 10 years of age: A prospective cohort study. Environ Int. 2018;113:259-68. . 10.1016/j.envint.2018.02.020 29459184

[11] Kippler M Tofail F Hamadani JD Gardner RM Grantham-McGregor SM Bottai M Early-Life Cadmium Exposure and Child Development in 5-Year-Old Girls and Boys: A Cohort Study in Rural Bangladesh. Environ Health Perspect. 2012;120:1462-8. . 10.1289/ehp.1104431 22759600 PMC3491924

[12] UNEP. An Assessment Report on Issues of Concern: Chemicals and Waste Issues Posing Risks to Human Health and the Environment. United Nations Environment Programme; 2020 Sep. Report No.: ISBN 978-92-807-3808-7|. Available: https://wedocs.unep.org/bitstream/handle/20.500.11822/33807/ARIC.pdf?sequence=1&isAllowed=y

[13] Fasinu P Orisakwe OE Heavy Metal Pollution in Sub-Saharan Africa and Possible Implications in Cancer Epidemiology. Asian Pac J Cancer Prev. 2013;14:3393-402. . 10.7314/APJCP.2013.14.6.3393 23886118

[14] Nwobi NL Owolabi JO Sotunsa JO Nwobi JC Abiodun RA Anetor JI Environmental cadmium exposure: a possible factor in the pathogenesis of preeclampsia. Int J Res Med Sci. 2019;7:4818-21. . 10.18203/2320-6012.ijrms20195563

[15] Anetor JI Rising Environmental Cadmium Levels in Developing Countries: Threat to Genome Stability and Health. Niger J Physiol Sci. 2012;27:103-15. . 10.4172/2161-0525.1000140 23652223

[16] Swaddiwudhipong W Mahasakpan P Bph TF Limpatanachote P Changes in Cadmium Exposure among Persons Living in Cadmium-Contaminated Areas in Northwestern Thailand: A Five-Year Follow-Up. J Med Assoc Thai. 2010;93:1217. 20973327

[17] Fukagawa NK Ziska LH Rice: Importance for Global Nutrition. J Nutr Sci Vitaminol (Tokyo). 2019;65:S2-3. . 10.3177/jnsv.65.S2 31619630

[18] Bandara JMRS Senevirathna DMAN Dasanayake DMRSB Herath V Bandara JMRP Abeysekara T Chronic renal failure among farm families in cascade irrigation systems in Sri Lanka associated with elevated dietary cadmium levels in rice and freshwater fish (Tilapia). Environ Geochem Health. 2008;30:465-78. . 10.1007/s10653-007-9129-6 18200439

[19] Yu H-Y Ding X Li F Wang X Zhang S Yi J The availabilities of arsenic and cadmium in rice paddy fields from a mining area: The role of soil extractable and plant silicon. Environ Pollut. 2016;215:258-65. . 10.1016/j.envpol.2016.04.008 27209244

[20] Meharg AA Norton G Deacon C Williams P Adomako EE Price A Variation in Rice Cadmium Related to Human Exposure. Environ Sci Technol. 2013;47:5613-8. . 10.1021/es400521h 23668419

[21] Kippler M Goessler W Nermell B Ekström EC Lönnerdal B El Arifeen S Factors influencing intestinal cadmium uptake in pregnant Bangladeshi women—A prospective cohort study. Environ Res. 2009;109:914-21. . 10.1016/j.envres.2009.07.006 19646688

[22] Kippler M Ekström E-C Lönnerdal B Goessler W Åkesson A El Arifeen S Influence of iron and zinc status on cadmium accumulation in Bangladeshi women. Toxicol Appl Pharmacol. 2007;222:221-6. . 10.1016/j.taap.2007.04.009 17543360

[23] Davies S Briand V Accrombessi M Fievet N Le Bot B Durand S Pre-conception serum ferritin concentrations are associated with metal concentrations in blood during pregnancy: A cohort study in Benin. Environ Res. 2021;202:111629. . 10.1016/j.envres.2021.111629 34242675

[24] Adie GU Oyebade EO Atanda BM Preliminary Study of Heavy Metals in Low-Cost Jewelry Items Available in Nigerian Markets. J Health Pollut. 2020;10:201202. . 10.5696/2156-9614-10.28.201202 33324499 PMC7731499

[25] Murphy T Lim S Kim S Irvine K Chaiwat W Wilson K Metal Contamination in Low-Cost Jewelry and Toys in Cambodia. J Health Pollut. 2016;6:47-57. . 10.5696/2156-9614-6-11.47 30524797 PMC6221481

[26] Cui X-Y Li S-W Zhang S-J Fan Y-Y Ma LQ Toxic metals in children’s toys and jewelry: coupling bioaccessibility with risk assessment. Environ Pollut. 2015;200:77-84. . 10.1016/j.envpol.2015.01.035 25700334

[27] Liu J Li Y Li D Wang Y Wei S The burden of coronary heart disease and stroke attributable to dietary cadmium exposure in Chinese adults, 2017. Sci Total Environ. 2022;825:153997. . 10.1016/j.scitotenv.2022.153997 35202702

[28] Lebbie TS Moyebi OD Asante KA Fobil J Brune-Drisse MN Suk WA E-Waste in Africa: A Serious Threat to the Health of Children. Int J Environ Res Public Health. 2021;18:8488. . 10.3390/ijerph18168488 34444234 PMC8392572

[29] Baldé CP, D’Angelo E, Luda V, Deubzer O, Kuehr R. Global Transboundary E-waste Flows Monitor. 2022;2022:66.

[30] Isimekhai KA Garelick H Watt J Purchase D Heavy metals distribution and risk assessment in soil from an informal E-waste recycling site in Lagos State, Nigeria. Environ Sci Pollut Res Int. 2017;24:17206-19. . 10.1007/s11356-017-8877-9 28589272

[31] Grant K Goldizen FC Sly PD Brune M-N Neira M van den Berg M Health consequences of exposure to e-waste: a systematic review. Lancet Glob Health. 2013;1:e350-61. . 10.1016/S2214-109X(13)70101-3 25104600

[32] Heacock M Kelly CB Asante KA Birnbaum LS Bergman ÅL Bruné M-N E-Waste and Harm to Vulnerable Populations: A Growing Global Problem. Environ Health Perspect. 2016;124:550-5. . 10.1289/ehp.1509699 26418733 PMC4858409

[33] WHO. Exposure to cadmium: a major public health concern. Geneva, Switzerland: World Health Organization; 2019. Available: https://www.who.int/publications-detail-redirect/WHO-CED-PHE-EPE-19-4-3

[34] Cullen JT, Maldonado MT. Biogeochemistry of Cadmium and Its Release to the Environment. In: Sigel A, Sigel H, Sigel RK, editors. Cadmium: From Toxicity to Essentiality. Dordrecht: Springer Netherlands; 2013. pp. 31–62. 10.1007/978-94-007-5179-8_2 10.1007/978-94-007-5179-8_2 23430769

[35] Satarug S Moore MR Adverse Health Effects of Chronic Exposure to Low-Level Cadmium in Foodstuffs and Cigarette Smoke. Environ Health Perspect. 2004;112:1099-103. . 10.1289/ehp.6751 15238284 PMC1247384

[36] Adedeji OH Olayinka OO Oyebanji FF Assessment of traffic related heavy metals pollution of roadside soils in emerging urban centres in Ijebu-North Area of Ogun State, Nigeria. J Appl Sci Environ Manag. 2013;17:509-14.

[37] WHO. Health risks of heavy metals from long-range transboundary air pollution. Copenhagen; 2007 p. 144.

[38] Satarug S Vesey DA Gobe GC Health Risk Assessment of Dietary Cadmium Intake: Do Current Guidelines Indicate How Much is Safe? Environ Health Perspect. 2017;125:284-8. . 10.1289/EHP108 28248635 PMC5332171

[39] Kaji M. Itai-itai Disease: Lessons for the Way to Environmental Regeneration. Chapter 7. In: Fujigaki Y, editor. Lessons From Fukushima. Cham: Springer International Publishing; 2015. pp. 141–65. 10.1007/978-3-319-15353-7 10.1007/978-3-319-15353-7

[40] Yoshida F Hata A Tonegawa H Itai-Itai disease and the countermeasures against cadmium pollution by the Kamioka mine. Environ Econ Policy Stud. 1999;2:215-29. . 10.1007/BF03353912

[41] Jin L Yu J Zhang L Ren A Comparison of Plasma Concentrations of Mercury, Cadmium, and Arsenic among Women in 2005 and 2012 in a Historically Contaminated Area in China. Biol Trace Elem Res. 2020;198:380-9. . 10.1007/s12011-020-02075-1 32072446

[42] Simmons RW, Sukreeyapongse O, Noble AD, Chinabut N. Report of LDD-IWMI Land Zoning and Cd Risk Assessment Activities Undertaken in Phatat Pha Daeng and Mae Tao Mai Sub- districts, Mae Sot, Tak Province, Thailand. Page78.

[43] EU. Commission Regulation (EU) 2021/1323 of 10 August 2021 amending Regulation (EC) No 1881/2006 as regards maximum levels of cadmium in certain foodstuffs (Text with EEA relevance). OJ L Aug 10, 2021. Available: http://data.europa.eu/eli/reg/2021/1323/oj/eng

[44] EEA. Heavy metal emissions in Europe. 2019 [cited 4 Mar 2022]. Available: https://www.eea.europa.eu/ims/heavy-metal-emissions-in-europe

[45] WHO. WHO Framework Convention on Tobacco Control. Geneva, Switzerland: World Health Organization; 2003. Report No.: ISBN 9241591013. Available: http://www.who.int/fctc/text_download/en/

[46] Hoffman SJ Poirier MJP Katwyk SRV Baral P Sritharan L Impact of the WHO Framework Convention on Tobacco Control on global cigarette consumption: quasi-experimental evaluations using interrupted time series analysis and in-sample forecast event modelling. BMJ. 2019;365:l2287. . 10.1136/bmj.l2287 31217191 PMC6582266

[47] Ouzzani M Hammady H Fedorowicz Z Elmagarmid A Rayyan—a web and mobile app for systematic reviews. Syst Rev. 2016;5:210. . 10.1186/s13643-016-0384-4 27919275 PMC5139140

[48] Page MJ McKenzie JE Bossuyt PM Boutron I Hoffmann TC Mulrow CD The PRISMA 2020 statement: an updated guideline for reporting systematic reviews. BMJ. 2021;372. . 10.1136/bmj.n71 33782057 PMC8005924

[49] Meader N King K Llewellyn A Norman G Brown J Rodgers M A checklist designed to aid consistency and reproducibility of GRADE assessments: development and pilot validation. Syst Rev. 2014;3:82. . 10.1186/2046-4053-3-82 25056145 PMC4124503

[50] Kelishadi R Hasanghaliaei N Poursafa P Keikha M Ghannadi A Yazdi M A randomized controlled trial on the effects of jujube fruit on the concentrations of some toxic trace elements in human milk. J Res Med Sci. 2016;21:108. . 10.4103/1735-1995.193499 28250785 PMC5322685

[51] Savchenko OV Bлaдимиpoвнa CO HEAVY METALS CLEARANCE WITH USE OF CALCIUM ALGINATE. Ekol Cheloveka Hum Ecol. 2014;21:20-4. . 10.17816/humeco17209

[52] Beletskaya EN Onul NM Glavatskaya VI Antonova EV Golovkova TA Clinical hygienic substantiation for the individual biocorrection of ecologically dependent conditions in the critical population groups industrial areas of Ukraine. Gig Sanit. 2014;1:64-7. 24749285

[53] Bisanz JE Enos MK Mwanga JR Changalucha J Burton JP Gloor GB Randomized Open-Label Pilot Study of the Influence of Probiotics and the Gut Microbiome on Toxic Metal Levels in Tanzanian Pregnant Women and School Children. MBio. 2014;5:e01580-14. . 10.1128/mBio.01580-14 25293764 PMC4196227

[54] El-Soud NHA Mohsen MA Joussef M Kazem Y Effect of a 2-Month Program of Antioxidants-Micronutrient-Rich Diet on Concentrations of Lead, Cadmium and Aluminum in Obese Egyptian Children. Maced J Med Sci. 2011;4:290-5. . 10.3889/MJMS.1857-5773.2011.0184

[55] Shafiei L Taymoori P Maleki A Nouri B Effect of Environmental Intervention on the Consumption of Rice without Toxic Metals Based on the Health Belief Model and Ecological-Social Model. J Clin Diagn Res. 2017;11:JC01-06. . 10.7860/JCDR/2017/26784.10262 28892931 PMC5583945

[56] Luzhetsky KP Ustinova OY Goleva OI Shtina IE Analysis of the effectiveness of technologies for correcting disorders of the physical development in children living in low-level atmospheric air pollution and drinking water with metals (lead, manganese, nickel, chromium, cadmium). Hyg Sanit. 2018;97:75-81. . 10.18821/0016-9900-2018-97-1-75-81

[57] Blaucok-Busch E Amin OR Dessoki HH Rabah T Efficacy of DMSA Therapy in a Sample of Arab Children with Autistic Spectrum Disorder. Maedica (Bucur). 2012;7:214-21. 23400264 PMC3566884

[58] Nawab J Ghani J Khan S Xiaoping W Minimizing the risk to human health due to the ingestion of arsenic and toxic metals in vegetables by the application of biochar, farmyard manure and peat moss. J Environ Manage. 2018;214:172-83. . 10.1016/j.jenvman.2018.02.093 29525749

[59] Khan S Reid BJ Li G Zhu Y-G Application of biochar to soil reduces cancer risk via rice consumption: A case study in Miaoqian village, Longyan, China. Environ Int. 2014;68:154-61. . 10.1016/j.envint.2014.03.017 24727070

[60] Khan MA Ding X Khan S Brusseau ML Khan A Nawab J The influence of various organic amendments on the bioavailability and plant uptake of cadmium present in mine-degraded soil. Sci Total Environ. 2018;636:810-7. . 10.1016/j.scitotenv.2018.04.299 29727847 PMC6063314

[61] Taghipour M Jalali M Effects of some industrial and organic wastes application on growth and heavy metal uptake by tomato (Lycopersicum esculentum) grown in a greenhouse condition. Environ Sci Pollut Res Int. 2020;27:5353-66. . 10.1007/s11356-019-07017-6 31848964

[62] Wang L Yang D Li Z Fu Y Liu X Brookes PC A comprehensive mitigation strategy for heavy metal contamination of farmland around mining areas – Screening of low accumulated cultivars, soil remediation and risk assessment. Environ Pollut. 2019;245:820-8. . 10.1016/j.envpol.2018.11.062 30502711

[63] da Silva WR da Silva FBV Araújo PRM do Nascimento CWA Assessing human health risks and strategies for phytoremediation in soils contaminated with As, Cd, Pb, and Zn by slag disposal. Ecotoxicol Environ Saf. 2017;144:522-30. . 10.1016/j.ecoenv.2017.06.068 28675866

[64] Barn P Gombojav E Ochir C Laagan B Beejin B Naidan G The effect of portable HEPA filter air cleaners on indoor PM2.5 concentrations and second hand tobacco smoke exposure among pregnant women in Ulaanbaatar, Mongolia: The UGAAR randomized controlled trial. Sci Total Environ. 2018;615:1379-89. . 10.1016/j.scitotenv.2017.09.291 29751442

[65] Brehmer C Norris C Barkjohn KK Bergin MH Zhang J Cui X The impact of household air cleaners on the oxidative potential of PM2.5 and the role of metals and sources associated with indoor and outdoor exposure. Environ Res. 2020;181:108919. . 10.1016/j.envres.2019.108919 31753466

[66] Brehmer C Norris C Barkjohn KK Bergin MH Zhang J Cui X The impact of household air cleaners on the chemical composition and children’s exposure to PM2.5 metal sources in suburban Shanghai. Environ Pollut. 2019;253:190-8. . 10.1016/j.envpol.2019.07.003 31310869

[67] Weidenhamer JD Fitzpatrick MP Biro AM Kobunski PA Hudson MR Corbin RW Metal exposures from aluminum cookware: An unrecognized public health risk in developing countries. Sci Total Environ. 2017;579:805-13. . 10.1016/j.scitotenv.2016.11.023 27866735

[68] Wang C Duan H-Y Teng J-W Assessment of Microwave Cooking on the Bioaccessibility of Cadmium from Various Food Matrices Using an In Vitro Digestion Model. Biol Trace Elem Res. 2014;160:276-84. . 10.1007/s12011-014-0047-z 24958019

[69] Shariatifar N Rezaei M Alizadeh Sani M Alimohammadi M Arabameri M Assessment of Rice Marketed in Iran with Emphasis on Toxic and Essential Elements; Effect of Different Cooking Methods. Biol Trace Elem Res. 2020;198:721-31. . 10.1007/s12011-020-02110-1 32189243

[70] Zhuang P Zhang C Li Y Zou B Mo H Wu K Assessment of influences of cooking on cadmium and arsenic bioaccessibility in rice, using an in vitro physiologically-based extraction test. Food Chem. 2016;213:206-14. . 10.1016/j.foodchem.2016.06.066 27451173

[71] Naseri M Rahmanikhah Z Beiygloo V Ranjbar S Effects of Two Cooking Methods on the Concentrations of Some Heavy Metals (Cadmium, Lead, Chromium, Nickel and Cobalt) in Some Rice Brands Available in Iranian Market. J Chem Health Risks. 2014;4. . 10.22034/jchr.2018.544068

[72] Díaz-Barriga F Batres L Calderón J Lugo A Galvao L Lara I The El Paso Smelter 20 Years Later: Residual Impact on Mexican Children. Environ Res. 1997;74:11-6. . 10.1006/enrs.1997.3741 9339209

[73] Cao S Duan X Ma Y Zhao X Qin Y Liu Y Health benefit from decreasing exposure to heavy metals and metalloid after strict pollution control measures near a typical river basin area in China. Chemosphere. 2017;184:866-78. . 10.1016/j.chemosphere.2017.06.052 28646769

[74] Jan FA Ishaq M Khan S Ihsanullah I Ahmad I Shakirullah M A comparative study of human health risks via consumption of food crops grown on wastewater irrigated soil (Peshawar) and relatively clean water irrigated soil (lower Dir). J Hazard Mater. 2010;179:612-21. . 10.1016/j.jhazmat.2010.03.047 20399016

[75] Campbell M McKenzie JE Sowden A Katikireddi SV Brennan SE Ellis S Synthesis without meta-analysis (SWiM) in systematic reviews: reporting guideline. BMJ. 2020;368:l6890. . 10.1136/bmj.l6890 31948937 PMC7190266

[76] European Commission. Cadmium. Cadmium in food. [cited 12 Aug 2022]. Available: https://food.ec.europa.eu/safety/chemical-safety/contaminants/catalogue/cadmium_en

[77] Hale S. ZeroPM EU project: About. In: ZeroPM [Internet]. 2022 [cited 15 Aug 2022]. Available: https://zeropm.eu/about/

[78] Gibb HJ Barchowsky A Bellinger D Bolger PM Carrington C Havelaar AH Estimates of the 2015 global and regional disease burden from four foodborne metals – arsenic, cadmium, lead and methylmercury. Environ Res. 2019;174:188-94. . 10.1016/j.envres.2018.12.062 30981404

[79] Liu K Zheng J Chen F Effects of washing, soaking and domestic cooking on cadmium, arsenic and lead bioaccessibilities in rice. J Sci Food Agric. 2018;98:3829-35. . 10.1002/jsfa.8897 29363749

[80] Pogoson E Carey M Meharg C Meharg AA Reducing the cadmium, inorganic arsenic and dimethylarsinic acid content of rice through food-safe chemical cooking pre-treatment. Food Chem. 2021;338:127842. . 10.1016/j.foodchem.2020.127842 32822902

[81] Rahman H Carey M Hossain M Savage L Islam MR Meharg AA Modifying the Parboiling of Rice to Remove Inorganic Arsenic, While Fortifying with Calcium. Environ Sci Technol. 2019;53:5249-55. . 10.1021/acs.est.8b06548 30993982

[82] Ghoochani M Rastkari N Yunesian M Nabizadeh Nodehi R Mesdaghinia A Houshiarrad A What do we know about exposure of Iranians to cadmium? Findings from a systematic review. Environ Sci Pollut Res Int. 2018;25:1-11. . 10.1007/s11356-017-0863-8 29260468

[83] Goudarzi MA Parsaei P Nayebpour F Rahimi E Determination of mercury, cadmium and lead in human milk in Iran. Toxicol Ind Health. 2013;29:820-3. . 10.1177/0748233712445047 22534496

[84] Barański M Średnicka-Tober D Volakakis N Seal C Sanderson R Stewart GB Higher antioxidant and lower cadmium concentrations and lower incidence of pesticide residues in organically grown crops: a systematic literature review and meta-analyses. Br J Nutr. 2014;112:794-811. . 10.1017/S0007114514001366 24968103 PMC4141693

[85] Monachese M Burton JP Reid G Bioremediation and Tolerance of Humans to Heavy Metals through Microbial Processes: a Potential Role for Probiotics? Appl Environ Microbiol. 2012;78:6397-404. . Accessed Mar 4, 2022. 10.1128/AEM.01665-12 22798364 PMC3426676

[86] Brown MJ McLaine P Dixon S Simon PA Randomized, Community-Based Trial of Home Visiting to Reduce Blood Lead Levels in Children. Pediatrics. 2006;117:147-53. . 10.1542/peds.2004-2880 16396872

[87] Sessa F Polito R Monda V Scarinci A Salerno M Carotenuto M Effects of a Plastic-Free Lifestyle on Urinary Bisphenol A Levels in School-Aged Children of Southern Italy: A Pilot Study. Front Public Health. 2021;9:626070. . 10.3389/fpubh.2021.626070 33598445 PMC7882684

[88] Giovanoulis G Nguyen MA Arwidsson M Langer S Vestergren R Lagerqvist A Reduction of hazardous chemicals in Swedish preschool dust through article substitution actions. Environ Int. 2019;130:104921. . 10.1016/j.envint.2019.104921 31229872

[89] Nussbaumer-Streit B Yeoh B Griebler U Pfadenhauer LM Busert LK Lhachimi SK Household interventions for preventing domestic lead exposure in children. Cochrane Database Syst Rev. 2016;10:CD006047. . 10.1002/14651858.CD006047.pub5 27744650 PMC6461195

[90] Rafati Rahimzadeh M Rafati Rahimzadeh M Kazemi S Moghadamnia A Cadmium toxicity and treatment: An update. Caspian J Intern Med. 2017;8:135-45. . 10.22088/cjim.8.3.135 28932363 PMC5596182

[91] UNHCR. Realizing the rights of the child through a healthy environment. Sect. UN Human Rights Council (45th sess: 2020) UN; Oct 7, 2020. Available: https://digitallibrary.un.org/record/3888433

