# Supplementary material for: Interventions to reduce cadmium exposure in low- and middle-income countries during pregnancy and childhood: A systematic review
Source: J Glob Health. 2022 Nov 12;12:04089. doi: 10.7189/jogh.12.04089 (PMC9653536; doi:10.7189/jogh.12.04089)
Supplement: Online Supplementary Document [file jogh-12-04089-s001.zip › S1-Cd-supplementarymaterial-searchstrings-bibliography-persian-chinese.docx]

**ONLINE SUPPLEMENTARY DOCUMENT**

**Title:** Interventions to reduce cadmium exposure in low- and middle-income countries during pregnancy and childhood: a systematic review

**Authors:** Kam Sripada & Adrian Madsen Lager

**I. Search string results**

**PubMed:** ("child"[Title/Abstract] OR "children"[Title/Abstract] OR "infant"[Title/Abstract] OR "infants"[Title/Abstract] OR "prenatal"[Title/Abstract] OR "neonate"[Title/Abstract] OR "newborn"[Title/Abstract] OR "baby"[Title/Abstract] OR "babies"[Title/Abstract] OR "mother"[Title/Abstract] OR "mothers"[Title/Abstract] OR "toddler"[Title/Abstract] OR "pre-school"[Title/Abstract] OR "parent"[Title/Abstract] OR "pediatric"[Title/Abstract] OR "woman"[Title/Abstract] OR "women"[Title/Abstract] OR "pregnan*"[Title/Abstract]) AND "cadmium"[Title/Abstract] AND ("intervention"[Title/Abstract] OR "prevent*"[Title/Abstract] OR "reduction"[Title/Abstract] OR "protect*"[Title/Abstract] OR "policy"[Title/Abstract] OR "program"[Title/Abstract] OR "project"[Title/Abstract] OR "control"[Title/Abstract] OR "parenting program"[Title/Abstract] OR "health education"[Title/Abstract] OR "instruction"[Title/Abstract] OR "outreach"[Title/Abstract] OR "counseling"[Title/Abstract] OR "health practice"[Title/Abstract] OR "mitigat*"[Title/Abstract] OR "clean up"[Title/Abstract] OR "remediat*"[Title/Abstract])

**Scopus:** TITLE-ABS-KEY ( child  OR  children  OR  infant  OR  infants  OR  prenatal  OR  neonate  OR  newborn  OR  baby  OR  babies  OR  mother  OR  mothers  OR  toddler  OR  pre-school  OR  parent  OR  pediatric  OR  woman  OR  women  OR  pregnan*  AND  cadmium  AND  intervention  OR  prevent*  OR  reduction  OR  protect*  OR  policy  OR  program  OR  project  OR  control  OR  "parenting program"  OR  "health education"  OR  instruction  OR  outreach  OR  counseling  OR  "health practice"  OR  mitigat*  OR  "clean up"  OR  remediat* )  AND  ( LIMIT-TO ( DOCTYPE ,  "ar" )  OR  LIMIT-TO ( DOCTYPE ,  "re" ) )

**WebofScience:** TS= ((Child or Children  OR infant  OR infants  OR prenatal  OR neonate  OR newborn  OR baby  OR babies  OR mother  OR mothers  OR toddler  OR pre-school  OR parent  OR pediatric  OR woman  OR women  OR pregnan*)  AND (cadmium)  AND (intervention  OR prevent*  OR reduction  OR protect*  OR policy  OR program  OR project  OR control  OR "parenting program"  OR "health education"  OR instruction  OR outreach  OR counseling  OR "health practice"  OR mitigat*  OR "clean up"  OR remediat* ))

**Global Health Medicus**: tw:((tw:(child)) OR (tw:(children)) OR (tw:(infant)) OR (tw:(infants)) OR (tw:(prenatal)) OR (tw:(neonate)) OR (tw:(newborn)) OR (tw:(baby)) OR (tw:(babies)) OR (tw:(mother)) OR (tw:(mothers)) OR (tw:(toddler)) OR (tw:(pre-school)) OR (tw:(parent)) OR (tw:(pediatric)) OR (tw:(woman)) OR (tw:(women)) OR (tw:(pregnan*)) AND (tw:(cadmium)) AND (tw:(intervention)) OR (tw:(prevent*)) OR (tw:(reduction)) OR (tw:(protect*)) OR (tw:(policy)) OR (tw:(program)) OR (tw:(project)) OR (tw:(control)) OR (tw:("parenting program")) OR (tw:("health education")) OR (tw:(instruction)) OR (tw:(outreach)) OR (tw:(counseling)) OR (tw:("health practice")) OR (tw:(mitigat*)) OR (tw:("clean up")) OR (tw:(remediat*)))

**Greenfile:** AB ( (child or children or infant or infants or prenatal or neonate or newborn or baby or babies or mother or mothers or toddler or pre-school or parent or pediatric or woman or women or pregnan*) ) AND AB ( (PFOA or PFAS or PFASs or PFBS or PFNA or PFHxA or PFCs or "Per- and polyfluoralkyl substances" or perfluorochemical* or "Perflourinated compund*") ) AND AB ( (intervention or prevent* or reduction or protect* or policy or program or project or control or "parenting program" or "health education" or instruction or outreach or counseling or "health practice" or mitigat* or "clean up" or remediat*) ) NOT TI ( (rat* or mouse or mice or murine or animal or chick or chicken or rabbit* or *fish or cell* or "in vivo" or "in vitro" or daphnia) )

*Search string results*

Scopus = 2466 results

WebofScience = 1596 results

Global Health Medicus = 48

PubMed = 851

Greenfile = 200

|  | Standard | Hand search | Total |
| --- | --- | --- | --- |
| Articles found | 5161 | 870 | 6031 |
| Duplicates removed | 1919 | 14 | 1933 |
| Articles for abstract/title screening | 3242 | 856 | 4098 |
| Articles removed through abstract screening | 3189 | 70 | 3259 |
| Articles for full text screening | 53 | 7 | 60 |
| Articles removed through full text screening | 34 | 0 | 34 |
| Articles for extraction | 19 | 7 | 26 |

**II. Bibliography of all included articles in systematic review**

1. Barn, P., Gombojav, E., Ochir, C., Laagan, B., Beejin, B., Naidan, G., Boldbaatar, B., Galsuren, J., Byambaa, T., Janes, C., Janssen, P. A., Lanphear, B. P., Takaro, T., Venners, S. A., Webster, G. M., Yuchi, W., Palmer, C. D., Parsons, P. J., Roh, Y. M., & Allen, R. W. (2018). The effect of portable HEPA filter air cleaners on indoor PM2.5 concentrations and second hand tobacco smoke exposure among pregnant women in Ulaanbaatar, Mongolia: The UGAAR randomized controlled trial. Science of the Total Environment, 615, 1379-1389., doi: 10.1016/j.scitotenv.2017.09.291
2. Beletskaya, E. N., Onul, N. M., Glavatskaya, V. I., Antonova, E. V., & Golovkova, T. A. (2014). Clinical and hygienic substantiation for the individual biocorrection of ecologically dependent conditions in the critical population groups industrial areas of Ukraine. Gigiena i sanitariia(1), 64-67.
3. Bisanz, J. E., Enos, M. K., Mwanga, J. R., Changalucha, J., Burton, J. P., Gloor, G. B., & Reid, G. (2014). Randomized Open-Label pilot study of the influence of probiotics and the gut microbiome on toxic metal levels in Tanzanian pregnant women and school children. mBio, 5(5)., doi: 10.1128/mBio.01580-14
4. Blaucok-Busch, E., Amin, O. R., Dessoki, H. H., & Rabah, T. (2012). Efficacy of DMSA Therapy in a Sample of Arab Children with Autistic Spectrum Disorder. Maedica (Bucur), 7(3), 214-221., PMID: 23400264
5. Brehmer, C., Norris, C., Barkjohn, K. K., Bergin, M. H., Zhang, J., Cui, X., Teng, Y., Zhang, Y., Black, M., Li, Z., Shafer, M. M., & Schauer, J. J. (2020). The impact of household air cleaners on the oxidative potential of PM2.5 and the role of metals and sources associated with indoor and outdoor exposure. Environmental Research, 181., doi: 10.1016/j.envres.2019.108919
6. Brehmer, C., Norris, C., Barkjohn, K. K., Bergin, M. H., Zhang, J., Cui, X., Zhang, Y., Black, M., Li, Z., Shafer, M., Schauer, J. J., The impact of household air cleaners on the chemical composition and children's exposure to PM2.5 metal sources in suburban Shanghai, Environmental Pollution, Volume 253, 2019, Pages 190-198, ISSN 0269-7491, doi: 10.1016/j.envpol.2019.07.003.
7. Cao, S., Duan, X., Ma, Y., Zhao, X., Qin, Y., Liu, Y., Li, S., Zheng, B., & Wei, F. (2017). Health benefit from decreasing exposure to heavy metals and metalloid after strict pollution control measures near a typical river basin area in China. Chemosphere, 184, 866-878., doi: 10.1016/j.chemosphere.2017.06.052
8. da Silva, W. R., da Silva, F. B. V., Araujo, P. R. M., & do Nascimento, C. W. A. (2017). Assessing human health risks and strategies for phytoremediation in soils contaminated with As, Cd, Pb, and Zn by slag disposal. Ecotoxicology and Environmental Safety, 144, 522-530., doi: 10.1016/j.ecoenv.2017.06.068
9. DiazBarriga, F., Batres, L., Calderon, J., Lugo, A., Galvao, L., Lara, I., Rizo, P., Arroyave, M. E., & McConnell, R. (1997). The El Paso smelter 20 years later: Residual impact on Mexican children. Environmental Research, 74(1), 11-16. doi: 10.1006/enrs.1997.3741
10. El-Soud, N. H. A., Mohsen, M. A., Joussef, M., & Kazem, Y. (2011). Effect of a 2-month program of antioxidants-micronutrient- rich diet on concentrations of lead, cadmium and aluminum in obese Egyptian children. Macedonian Journal of Medical Sciences, 4(3), 290-295., doi: 10.3889/MJMS.1857-5773.2011.0184
11. Jan, F. A., Ishaq, M., Khan, S., Ihsanullah, I., Ahmad, I., Shakirullah, M., A comparative study of human health risks via consumption of food crops grown on wastewater irrigated soil (Peshawar) and relatively clean water irrigated soil (lower Dir). J Hazard Mater. 2010 Jul 15;179(1-3):612-21., doi: 10.1016/j.jhazmat.2010.03.047.
12. Jin, L., Yu, J. R., Zhang, L., & Ren, A. G. (2020). Comparison of Plasma Concentrations of Mercury, Cadmium, and Arsenic among Women in 2005 and 2012 in a Historically Contaminated Area in China. Biological Trace Element Research, 198(2), 380-389., doi: 10.1007/s12011-020-02075-1
13. Kelishadi, R., Hasanghaliaei, N., Poursafa, P., Keikha, M., Ghannadi, A., Yazdi, M., & Rahimi, E. (2016). A randomized controlled trial on the effects of jujube fruit on the concentrations of some toxic trace elements in human milk. Journal of Research in Medical Sciences, 21(8)., doi: 10.4103/1735-1995.193499
14. Khan, M. A., Ding, X., Khan, S., Brusseau, M. L., Khan, A., Nawab, J., The influence of various organic amendments on the bioavailability and plant uptake of cadmium present in mine-degraded soil. Sci Total Environ. 2018 Sep 15;636:810-817., doi: 10.1016/j.scitotenv.2018.04.299.
15. Khan S, Reid BJ, Li G, Zhu YG. Application of biochar to soil reduces cancer risk via rice consumption: a case study in Miaoqian village, Longyan, China. Environ Int. 2014 Jul;68:154-61., doi: 10.1016/j.envint.2014.03.017.
16. Luzhetsky, K. P., Ustinova, O. Y., Goleva, O. I., & Shtina, I. E. (2018). Analysis of the effectiveness of technologies for correcting disorders of the physical development in children living in low-level atmospheric air pollution and drinking water with metals (Lead Manganese, Nickel, Chromium, Cadmium). Gigiena i Sanitariya, 97(1), 75-81.
17. Naseri, M., Rahmanikhah, Z., Beiygloo, V., Ranjbar, S. (2018). Effects of Two Cooking Methods on the Concentrations of Some Heavy Metals (Cadmium, Lead, Chromium, Nickel and Cobalt) in Some Rice Brands Available in Iranian Market. Journal of Chemical Health Risks, 4(2)., doi: 10.22034/jchr.2018.544068
18. Nawab, J., Ghani, J., Khan, S., & Xiaoping, W. (2018). Minimizing the risk to human health due to the ingestion of arsenic and toxic metals in vegetables by the application of biochar, farmyard manure and peat moss. Journal of Environmental Management, 214, 172-183., doi: 10.1016/j.jenvman.2018.02.093
19. Savchenko, O. V. (2014). Heavy metals clearance with use of calcium alginate. Human Ecology (Russian Federation)(8), 20-24., doi: 10.33396/1728-0869-2014-8-20-24
20. Shafiei, L., Taymoori, P., Maleki, A., & Nouri, B. (2017). Effect of Environmental Intervention on the Consumption of Rice without Toxic Metals Based on the Health Belief Model and Ecological-Social Model. Journal of Clinical and Diagnostic Research, 11(7), JC01-JC06., doi: 10.7860/JCDR/2017/26784.10262
21. Shariatifar, N., Rezaei, M., Alizadeh Sani, M., Alimohammadi, M., & Arabameri, M. (2020). Assessment of Rice Marketed in Iran with Emphasis on Toxic and Essential Elements; Effect of Different Cooking Methods. Biological Trace Element Research, 198(2), 721-731., doi: 10.1007/s12011-020-02110-1
22. Taghipour, M., & Jalali, M. (2020). Effects of some industrial and organic wastes application on growth and heavy metal uptake by tomato (Lycopersicum esculentum) grown in a greenhouse condition. Environmental Science and Pollution Research, 27(5), 5353-5366., doi: 10.1007/s11356-019-07017-6
23. Wang, C., Duan, H. Y., Teng, J. W., Assessment of microwave cooking on the bioaccessibility of cadmium from various food matrices using an in vitro digestion model. Biol Trace Elem Res. 2014 Aug;160(2):276-84., doi: 10.1007/s12011-014-0047-z
24. Wang, L., Yang, D., Li, Z., Fu, Y., Liu, X., Brookes, P. C., & Xu, J. (2019). A comprehensive mitigation strategy for heavy metal contamination of farmland around mining areas – Screening of low accumulated cultivars, soil remediation and risk assessment. Environmental Pollution, 245, 820-828., doi: 10.1016/j.envpol.2018.11.062
25. Weidenhamer, J. D., Fitzpatrick, M. P., Biro, A. M., Kobunski, P. A., Hudson, M. R., Corbin, R. W., Gottesfeld, P., Metal exposures from aluminum cookware: An unrecognized public health risk in developing countries. Sci Total Environ. 2017 Feb 1;579:805-813., doi: 10.1016/j.scitotenv.2016.11.023.
26. Zhuang, P., Zhang, C., Li, Y., Zou, B., Mo, H., Wu, K., Wu, J., Li, Z., Assessment of influences of cooking on cadmium and arsenic bioaccessibility in rice, using an in vitro physiologically-based extraction test. Food Chem. 2016 Dec 15;213:206-214., doi: 10.1016/j.foodchem.2016.06.066.

**III. Abstract in Persian**

چکیده

زمینه

در معرض قرار گرفتن با فلز سمی کادمیوم در سطح جهانی گسترده و به ویژه در کشورهای با درآمد کم و متوسط (LMICs)

​​ شایع است. اوایل زندگی (از بارداری تا دوران کودکی) پنجره ای آسیب پذیر برای در معرض قرار گرفتن است. بنابراین، مداخله سلامت درقوانین کشورهای با درآمد کم و متوسط ​​برای جلوگیری یا کاهش مواجهه اولیه با کادمیم ممکن است در بهبود سلامت عمومی مرتبط باشد.

روش ها

این مقاله مروری سیستماتیک از 5 پایگاه داده Scopus، Web of Science، Global Health Medicus، Greenfile و PubMed می باشد که در مجموع 4098 مقاله یافته شده که از این تعداد 26 مورد مرتبط و برای این بررسی استخراج شده است. به دلیل ناهمگونی گسترده مطالعات در برگیرنده، سنتز بدون متاآنالیز (سنتز روایت) برای تجزیه و تحلیل داده ها استفاده شده است. کیفیت مطالعه و خطر سوگیری با بکارگیری معیارهای GRADE تغییر یافته ارزیابی شده است.

نتایج

در مجموع 26 مطالعه از 21 LMIC شناسایی شده که محدودی ای از سیاست‌ها گرفته تا درمان بالینی، روش‌های توانبخشی و پاک‌سازی خاک کشاورزی، مداخلات سلامت در تغذیه و پخت و پز، و استراتژی‌های ضد آلودگی در سطح خانوار را شامل می شود. مداخلات سلامت در واقع کودکان، زنان باردار و پس از زایمان و/یا زنان در سنین باروری را هدف قرار دادند. در حالی که چندین مطالعه شواهدی از اثربخشی ارائه کردند، به نظر نمی رسید که هیچ یک راه حل واقع بینانه برای آلودگی کادمیوم در مقیاس بزرگ ارائه دهد. مطالعات در مورد کشاورزی و آماده سازی مواد غذایی ، به ویژه در مورد برنج نسبتاً مکرر بود. مطالعات فیلتراسیون هوا در دوران بارداری ، اثربخشی در کاهش قرار گرفتن در معرض کادمیم در محیط درونی ساختمان را نشان می دهد.

نتیجه گیری

آلودگی کادمیوم یک تهدید دائمی و گسترده برای سلامت کودکان با راه حل های بسیار کمی است. آسیب طولانی ‌مدت به سلامت کودکان که از سال‌های اولیه شروع می‌شود، باید انگیزه سرمایه‌گذاری در مداخلات سلامت، نوآوری‌ها و تحقیقات بیشتر با کیفیت بالاتر باشد.

ثبت

روش های مرور سیستماتیک با PROSPERO (CRD42021235435) ثبت شه است.

[متن کامل مقاله به زبان انگلیسی موجود است.]

**III. Abstract in Chinese**

**摘要 (Abstract)**

**背景**

暴露于有毒金属镉的现象在全球范围内普遍存在，尤其是在中低收入国家。生命早期（从怀孕到儿童时期）是镉暴露的敏感窗口期。因此，在中低收入国家采取干预措施，防止或减少生命早期对镉(Cd)的接触，可能对改善公共健康有意义。本文就中低收入国家的镉暴露的干预研究进行了系统性回顾。

**研究方法**

**从**Scopus、Web of Science、Global Health Medicus、Greenfile 和 PubMed这五个数据库进行系统性文献搜寻，我们共发现了4098 篇文章。其中有26篇文章与本综述相关并被提取。由于所纳入的研究具有广泛的异质性，因此在数据分析中采用了无荟萃分析的综合方法（叙述性综合）。使用修正后的GRADE标准来评估研究质量和偏倚风险。

**结果**

共确定了来自21个中低收入国家的 26 项研究，这些研究涉及的范围包括从政策到临床治疗、农业土壤的恢复和清理方法、营养和烹饪干预以及家庭层面的抗污染策略。这些干预措施主要针对儿童、孕妇和产后妇女和/或育龄妇女。虽然有几项研究提供了一些有效性的证据，但似乎都没有为大规模镉污染提供一个切实可行的解决方案。关于农业和食品制备的研究相对较多，特别是与大米有关的研究。另外一些研究表明在怀孕期间使用空气净化器对减少室内镉暴露有一定的效果。

**结论**

镉污染是对儿童健康的一个持续而广泛的威胁，但确定的解决镉污染的方案太少。镉暴露对儿童从幼年开始的长期健康损害应促使人们投资于更高质量的干预措施、创新和进一步研究。

**注册**

本系统评价已在国际化前瞻性注册数据库（PROSPERO）(CRD42021235435)注册。

[文章全文请见英文版]
